# High-Sensitivity AlGaN/GaN HFET Implantable Neural Probe Without Gate Control Enabled by Photoelectrochemical Etching

**DOI:** 10.3390/mi17080956

**Published:** 2026-08-12

**Authors:** Yanyuan Ding, Xin Cao, Yang Li, Xien Yang, Ye Wen, Xiaodong Li, Xilei Huang, Zeyi Li, Jiefeng Weng, Baijun Zhang

**Affiliations:** State Key Laboratory of Optoelectronic Materials and Technologies, School of Electronics and Information Technology, Sun Yat-sen University, Guangzhou 510006, China; dingyy27@mail2.sysu.edu.cn (Y.D.); caox58@mail2.sysu.edu.cn (X.C.); liyang325@mail2.sysu.edu.cn (Y.L.); yangxen@mail2.sysu.edu.cn (X.Y.); weny87@mail2.sysu.edu.cn (Y.W.); lixd65@mail2.sysu.edu.cn (X.L.); huangxlei@mail2.sysu.edu.cn (X.H.); lizy298@mail2.sysu.edu.cn (Z.L.); wengjf3@mail2.sysu.edu.cn (J.W.)

**Keywords:** neuroelectrophysiology, AlGaN/GaN heterojunction field-effect transistor, neural probe, photoelectrochemical etching

## Abstract

Implantable neural probes that can simultaneously possess biocompatibility, electrochemical stability, and high signal fidelity are the core devices in neuroelectrophysiological research. In this article, AlGaN/GaN heterojunction field-effect transistors are used instead of traditional metal microelectrodes to prepare brain nerve probes. By photoelectrochemical etching and optimization of sensing area size, the probes have the maximum transconductance value, i.e., the highest sensitivity, under no gate control. After digital filtering processing, the neural probe achieved a signal-to-noise ratio of 8.04 dB on biological analog signals as low as 50 µV, confirming its ability to detect microvolt-level signals. The ex vivo recording of the bullfrog sciatic nerve further validated its biosensing performance, demonstrating the selective capture of composite action potentials from active neural tissue.

## 1. Introduction

Implantable neural probes are critical tools for investigating neural circuits, diagnosing neurological disorders, and enabling emerging brain–computer interface technologies [1,2,3]. To achieve reliable neural recording in clinical and research applications, implantable interfaces must simultaneously provide high signal fidelity, long-term stability, minimal tissue response, and high recording density [4,5,6]. However, these requirements remain difficult to satisfy because conventional neural interfaces face intrinsic limitations associated with their signal transduction mechanisms and material properties [7].

Most existing implantable neural probes rely on metal microelectrodes, where neural signals are converted through charge transfer processes occurring at the metal–electrolyte interface. Although metal electrodes provide excellent conductivity and have been widely adopted for electrophysiological recording, their long-term performance is constrained by electrochemical instability, including interfacial polarization, impedance variation, and material degradation during chronic implantation [1,8]. These effects can introduce signal drift and increase the noise level over extended recording periods [6,9]. Meanwhile, advanced CMOS-based neural arrays have significantly improved recording density and signal processing capability through integrated amplification and multiplexing [4,10,11,12]. Nevertheless, these systems still fundamentally depend on electrode-based interfaces and typically require external reference electrodes to establish stable electrical potentials, limiting further miniaturization and long-term reliability [13,14].

Field-effect-transistor-based neural interfaces provide an alternative sensing strategy by replacing direct electrochemical charge transfer with semiconductor-based transduction [15,16,17]. In FET sensors, extracellular potential variations or surface charge changes modulate the channel conductivity, enabling direct electrical amplification of biological signals at the sensing interface [18,19,20]. This mechanism reduces reliance on direct electrochemical charge transfer by converting extracellular potential variations into semiconductor channel modulation and provides a scalable pathway toward high-density semiconductor neural interfaces [21]. Furthermore, the compatibility of FET devices with conventional semiconductor fabrication processes offers opportunities for monolithic integration with signal conditioning and readout circuits [22,23,24,25].

Among various FET platforms, AlGaN/GaN heterojunction field-effect transistors (HFETs) are particularly attractive for neural sensing applications due to their high electron mobility, large transconductance, wide operating range, and excellent chemical stability [26,27,28]. These characteristics provide strong potential for detecting weak extracellular electrophysiological signals. However, a fundamental challenge remains unresolved: conventional AlGaN/GaN HFETs typically exhibit depletion-mode characteristics, where the maximum transconductance occurs under a negative gate bias. Therefore, a reference electrode is generally required to apply and maintain the appropriate gate potential during biological sensing [18,29]. Although previous studies have explored reference-electrode-assisted AlGaN/GaN HFET biosensors, the additional electrode introduces extra material interfaces and potential instability, which compromises the advantages of semiconductor-based neural interfaces for chronic implantation [30].

Achieving reference-electrode-free operation requires precise modulation of the electrostatic coupling between the biological interface and the two-dimensional electron gas (2DEG) channel [31,32]. Early recessed-gate AlGaN/GaN devices demonstrated that controlled thinning of the AlGaN barrier can modify the 2DEG properties, threshold voltage, and transconductance [27,33,34,35]. In particular, Mikulics et al. showed that recessing an approximately 20 nm AlGaN barrier by about 6 nm shifted the gate voltage corresponding to the maximum transconductance from approximately −3 V to −0.2 V, while also modifying the residual strain in the heterojunction [36]. These studies established the device-physics basis for recess-controlled modulation of AlGaN/GaN HFETs. However, the use of such recess engineering to directly align the maximum-sensitivity operating point with zero gate bias for reference-electrode-free neural sensing remains insufficiently explored [32,33,37].

In this work, we develop a reference-electrode-free AlGaN/GaN HFET neural probe through synergistic device and surface engineering. A photoelectrochemical (PEC) etching strategy is first introduced to selectively oxidize and dissolve the exposed AlGaN barrier, forming a recessed sensing region that modifies the surface-to-channel electrostatic coupling and shifts the peak transconductance toward zero gate bias [34,38]. This PEC-enabled recess engineering eliminates the need for an external reference electrode and enables self-biased operation of the depletion-mode HFET. Furthermore, the sensing-region geometry is optimized by reducing the active area between the source and drain electrodes, which enhances intrinsic transconductance and improves signal amplification capability [39]. The optimized device achieves microvolt-level signal detection with improved signal-to-noise performance. Finally, ex vivo recording from bullfrog sciatic nerves validates the capability of the proposed HFET probe for detecting stimulus-evoked neural activity without a reference electrode.

## 2. Materials and Methods

### 2.1. Epitaxial Growth and Device Fabrication

**Figure 1:**
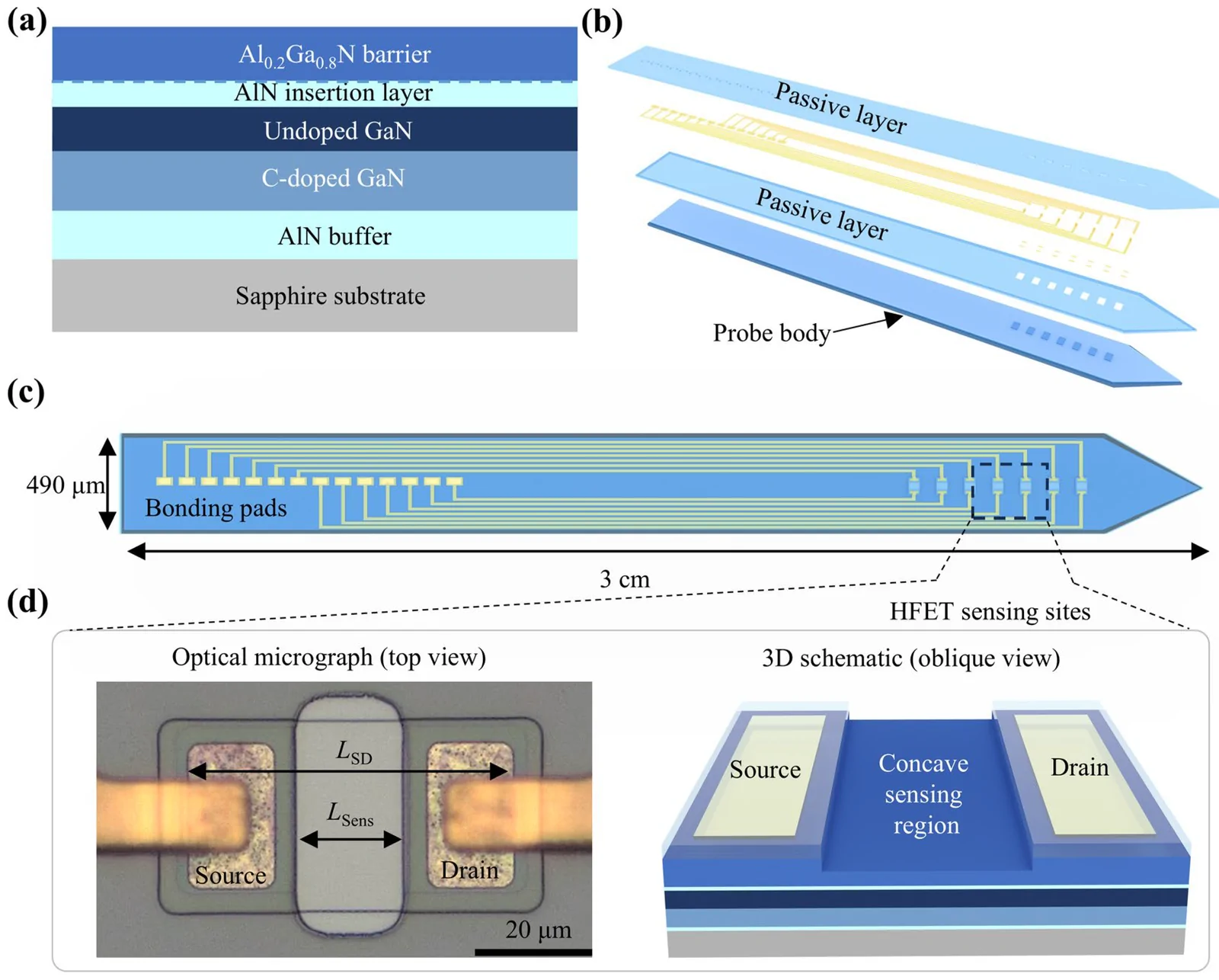
Structural design of the reference-electrode-free AlGaN/GaN HFET neural probe: (**a**) Epitaxial structure; (**b**) exploded view of the neural probe; (**c**) overall layout of the neural probe; (**d**) optical micrograph and 3D schematic of the recessed HFET sensing region.

In this study, the AlGaN/GaN heterojunction used for the concave HFET neural probe was grown on a c-plane sapphire substrate by metal–organic chemical vapor deposition (MOCVD), as shown in Figure 1a. From the substrate upward, the epitaxial stack consisted of 20 nm AlN buffer layer, 2 µm carbon-doped GaN buffer layer, 0.3 µm undoped GaN layer, 1 nm AlN insertion layer, and 25 nm Al_0.2_Ga_0.8_N barrier layer. Room-temperature four-probe Hall measurements performed using an ACCENT HL5500PC Hall system (Accent Optical Technologies, York, UK) showed a sheet carrier density of 1.026 × 10^13^ cm^−2^ and an electron mobility of 1210 cm^2^ V^−1^ s^−1^.

Using this MOCVD-grown heterojunction, the HFET neural probe was fabricated through a sequential microfabrication process, as shown in Figure 1b,c. Mesa isolation was first defined by inductively coupled plasma etching with Cl_2_/BCl_3_ chemistry down to the carbon-doped GaN buffer layer. A thin SiO_2_ passivation layer was then deposited by PECVD, and contact windows were opened using BOE. Source and drain ohmic contacts were formed by electron-beam evaporation of Ti/Al/Ni/Au, followed by rapid thermal annealing at 850 °C for 30 s in N_2_ to reduce contact resistance. Metal interconnects were subsequently deposited to connect the HFET sensing units to the bonding pads. Finally, 1 µm SiO_2_ passivation layer was deposited by PECVD, and the SiO_2_ above the sensing region was selectively etched to expose the AlGaN barrier for subsequent photoelectrochemical etching.

### 2.2. Photoelectrochemical Etching of the Recessed Sensing Region

**Figure 2:**
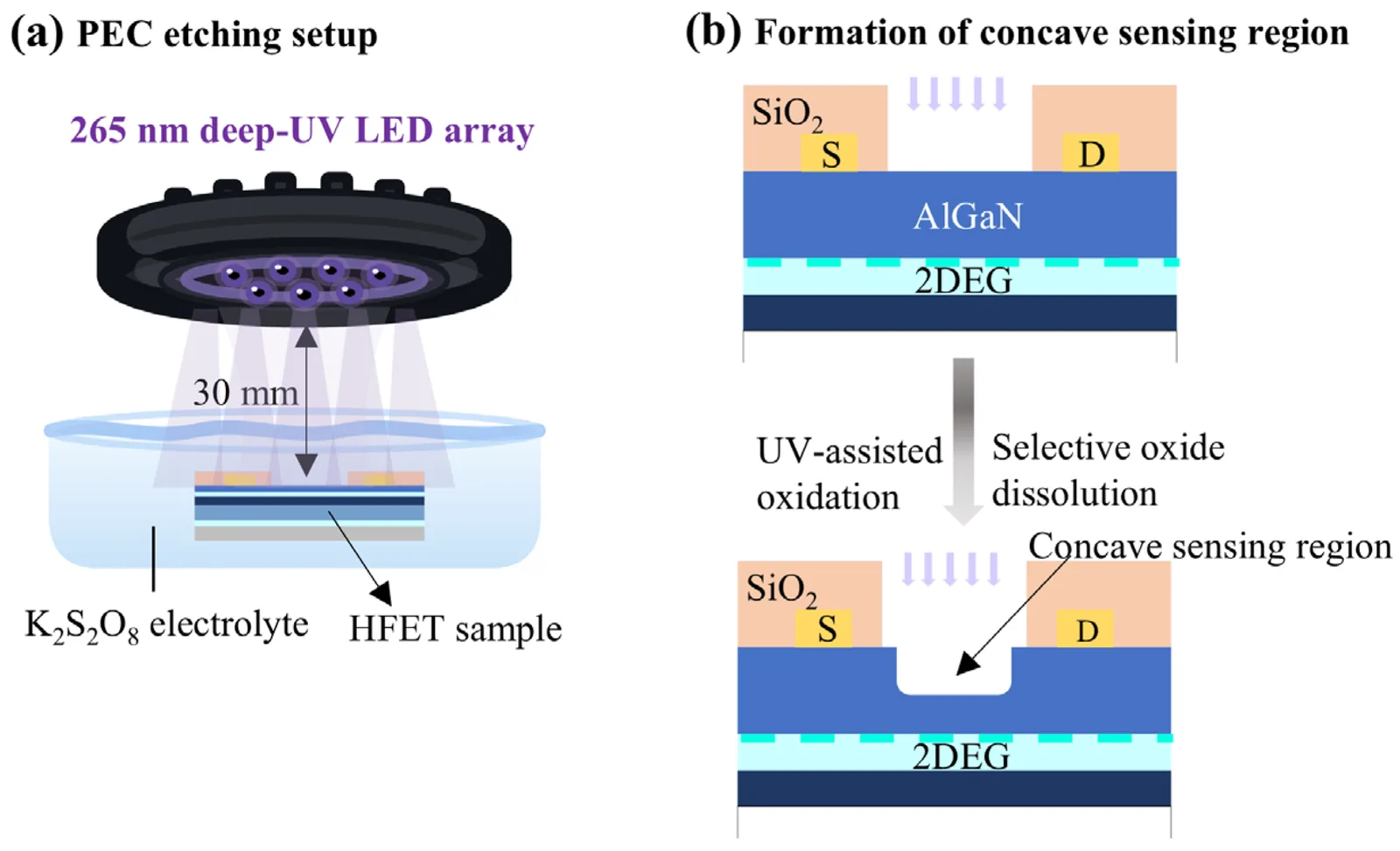
Formation of the concave sensing region by PEC etching: (**a**) Etching setup; (**b**) before and after PEC etching.

The recessed sensing region was formed by photoelectrochemical etching after completion of the passivated HFET structure. As shown in Figure 1d, this post-treatment created a concave sensing region designed to enhance the response of the device to local surface potential changes. The photoelectrochemical etching process, illustrated in Figure 2a, used a 265 nm deep-UV LED array with a power density of 40.0 mW/cm^2^ as the illumination source and a 0.52 mol/L K_2_S_2_O_8_ solution as the electrolyte.

During photoelectrochemical etching, UV illumination oxidized the exposed AlGaN barrier surface, as illustrated in Figure 2b. The generated oxide was then selectively dissolved in the K_2_S_2_O_8_ electrolyte, gradually thinning the exposed barrier region. This localized thinning produced the concave sensing recess while preserving the surrounding passivated device areas. The corresponding photoelectrochemical reaction is given in Equation (1) [38].

(1) $$\mathrm{AlGaN}+\mathrm{photo}\;\;\;{\mathrm{carries}(6h}^{+}+{6e}^{-})+{6\mathrm{SO}}_{4}^{-}\to {\mathrm{Al}}^{3+}+{\mathrm{Ga}}^{3+}+{6\mathrm{SO}}_{4}^{2-}+\frac{1}{2}N_{2}$$

### 2.3. Electrical Characterization and Signal Analysis

The passivated device was then characterized in PBS using a Agilent B1500A semiconductor parameter analyzer (Keysight Technologies, Santa Rosa, CA, USA). During testing, the source and drain electrodes were connected to the analyzer, which supplied the drain-source voltage, *V*_DS_. A small potential perturbation was applied to the sensing region through an external signal source to simulate interfacial potential changes associated with biomolecular or neural activity.

Several noise sources may contribute to the measured source-drain current fluctuations in this experimental configuration. First, the external signal generator, connectors, and measurement electronics introduce source and transmission-chain noise. Second, thermal noise arises from the HFET channel, ohmic contacts, interconnects, and associated measurement circuitry. In addition, low-frequency 1/f noise associated with carrier fluctuations and interface trapping in the AlGaN/GaN HFET may contribute to the baseline fluctuation, particularly within the low-frequency neural recording band. Environmental electromagnetic interference, including power-line and common-mode coupling, may also be introduced through the experimental wiring and measurement environment [14]. Therefore, the experimentally measured baseline noise represents the combined noise of the complete sensing and measurement system rather than the intrinsic noise of the HFET alone.

Finally, back-end signal processing was applied to all source-drain current recordings to improve the detectability of the stimulus-locked response against the combined system-level background noise. Digital filtering was used to preserve the amplitude and temporal morphology of the stimulus-locked response. The same signal-processing procedure was applied to both the stimulus–response and baseline segments before SNR evaluation.

(2) $$\mathrm{SNR}=20\cdot \log(\frac{S}{N})$$

Detectability was quantified using the post-filtered SNR in Equation (2). Here, *S* denotes the RMS value of the detected peak-to-peak response amplitudes within the stimulus-locked time window, and *N* denotes the RMS value of the baseline noise measured before stimulation.

## 3. Results and Discussion

### 3.1. Photoelectrochemical Tuning of Transconductance and Operating Point

Photoelectrochemical etching was first used to tune the electrical characteristics of the AlGaN/GaN HFET neural probe for reference-electrode-free operation. Because the device is designed to operate without an externally applied gate bias in physiological environments, its most sensitive operating point must be positioned near zero gate voltage.

**Figure 3:**
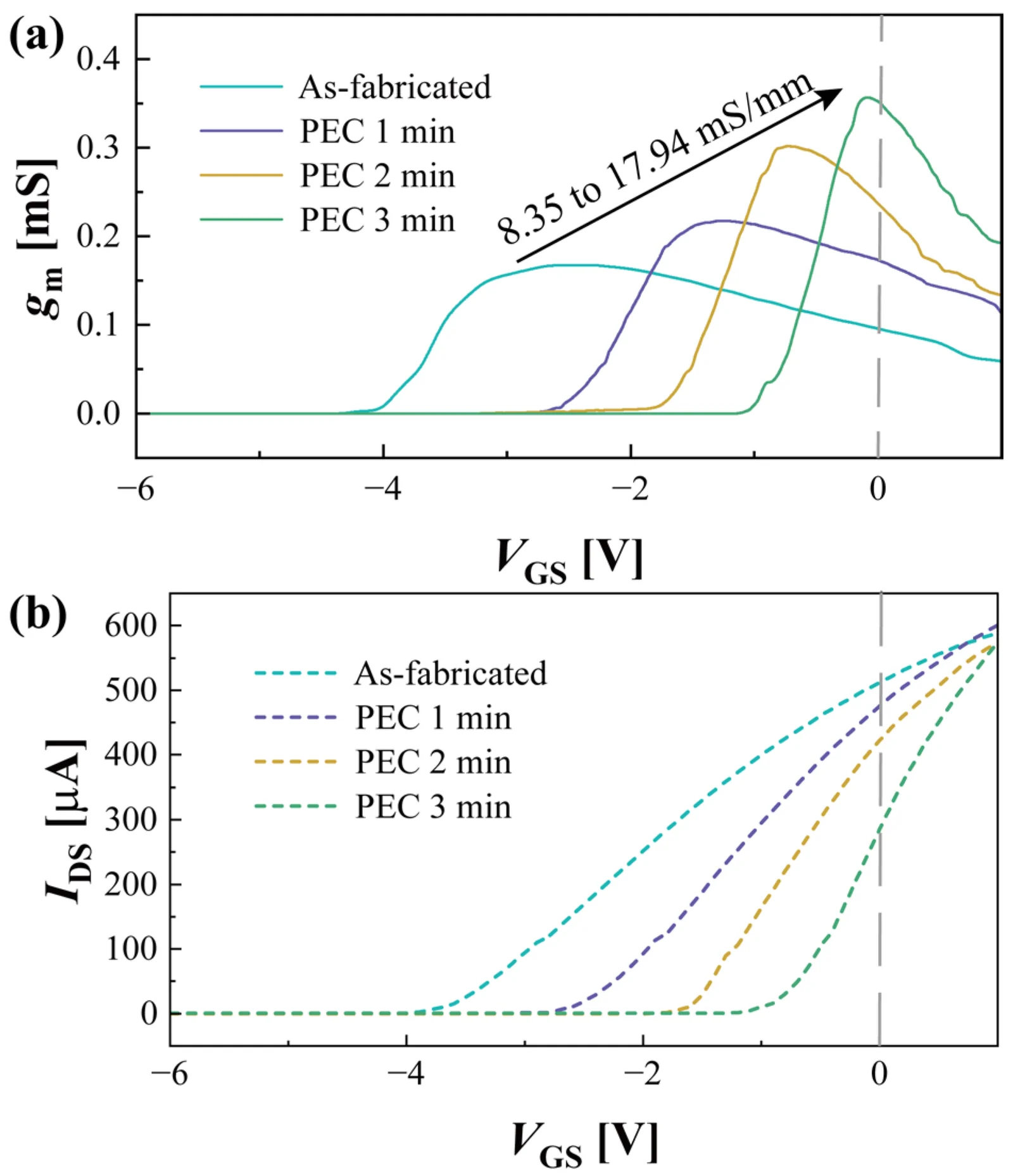
PEC tuning of AlGaN/GaN HFET characteristics: (**a**) *g*_m_-*V*_GS_ curves before and after PEC etching; (**b**) corresponding *I*_DS_-*V*_GS_ transfer curves.

As shown in Figure 3a,b, photoelectrochemical etching steepened the transfer characteristics, indicating that the source-drain current became more responsive to changes in gate voltage. This change was accompanied by a marked increase in peak transconductance, from 8.35 to 17.94 mS/mm, corresponding to an enhancement of more than 100%. At the same time, the transconductance peak shifted from the negative gate-voltage region toward zero bias. Time-dependent measurements showed that, after 3 min of etching, the peak transconductance reached approximately 17.94 mS/mm near 0 V. These results demonstrate that photoelectrochemical etching can both enhance device sensitivity and align the optimal operating point with the zero-gate-bias condition required for reference-electrode-free neural recording.

In addition to the electrostatic effect associated with AlGaN barrier thinning, the recessing process may also modify the local strain state of the AlGaN/GaN heterojunction. Previous micro-photoluminescence studies of recessed AlGaN/GaN HFETs have revealed spatially non-uniform strain distributions across the source–recess–drain region. Because the 2DEG density in AlGaN/GaN heterojunction is strongly influenced by spontaneous and piezoelectric polarization, local strain redistribution may consequently produce a non-uniform lateral distribution of sheet carrier density within the recessed sensing region. For the concave geometry employed here, variations in recess depth and strain may therefore introduce local variations in channel sheet resistance, particularly near the transition between recessed and unrecessed regions. Such variations could contribute to the effective source or drain access and channel resistance and thereby affect the measured transconductance.

### 3.2. Sensing-Region Geometry Optimization for Enhanced Transconductance

**Figure 4:**
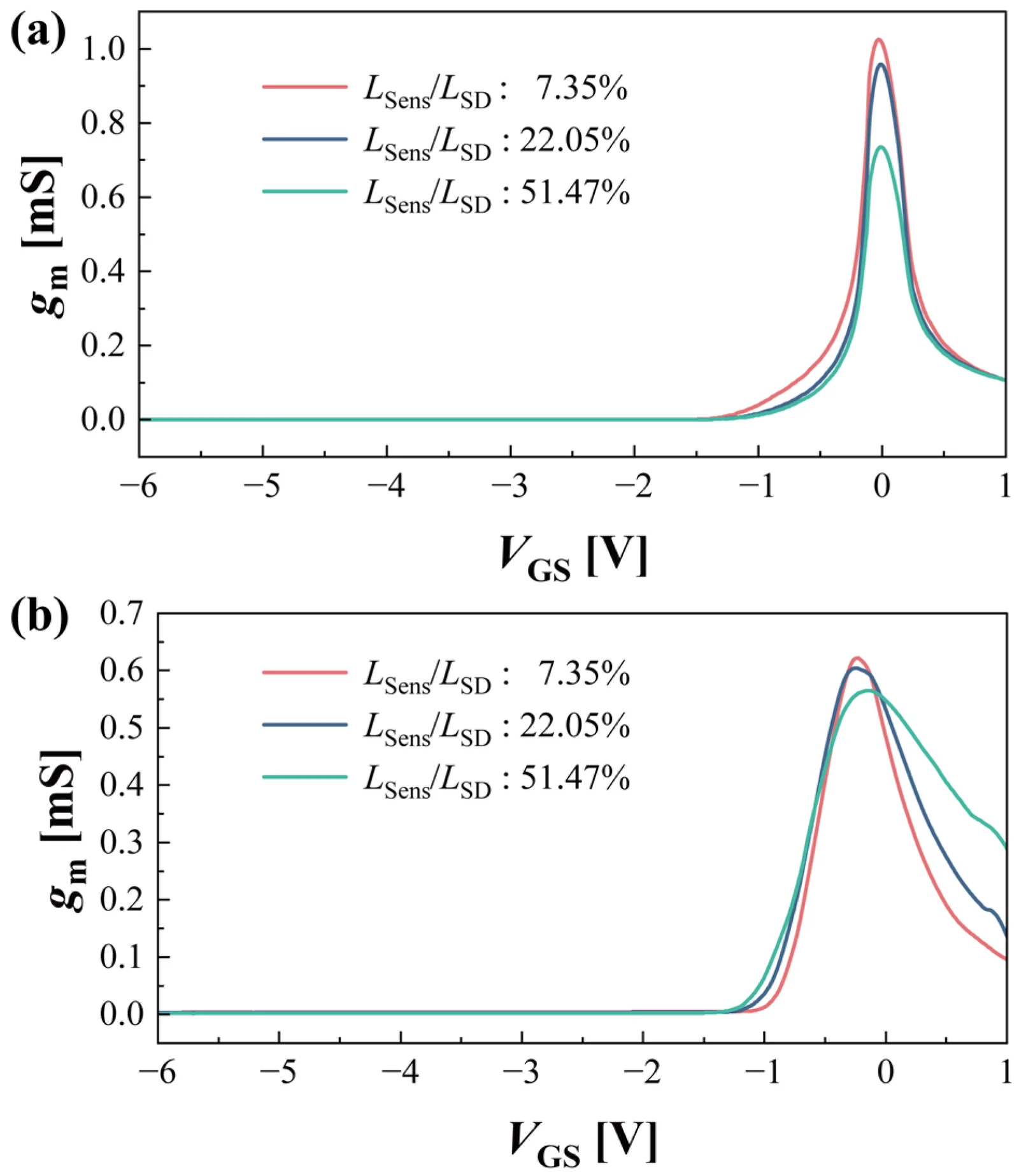
Effect of sensing-region geometry on transconductance: (**a**) Simulated *g*_m_-*V*_GS_ curves for different *L*_Sens_/*L*_SD_ ratios; (**b**) measured *g*_m_-*V*_GS_ curves of devices with corresponding sensing-region ratios.

Having established the role of photoelectrochemical tuning, we next examined whether channel geometry could further improve transconductance. In particular, we evaluated how the fraction of the source-drain channel occupied by the sensing region affects device performance. Sentaurus TCAD 2018.06 simulations, shown in Figure 4a, revealed that transconductance decreased monotonically as the sensing-region-length to source-drain-spacing ratio, *L*_Sens_/*L*_SD_, increased. This trend indicates that reducing the relative sensing-region fraction within the channel can increase transconductance and strengthen the device’s response to small electrical signals.

This geometry-dependent trend was then verified experimentally using AlGaN/GaN HFET neural probes with varying sensing-region lengths. As shown in Figure 4b, the measured results were consistent with the simulation, with devices containing a smaller sensing-region fraction exhibiting higher transconductance. By combining photoelectrochemical treatment with channel-geometry optimization, the peak transconductance was further increased to 32.47 mS/mm. This result confirms that the co-optimization strategy effectively improves the intrinsic sensitivity of the neural probe while maintaining operation near zero gate bias.

### 3.3. Reference-Electrode-Free Detection of Analog Neural Signals

**Figure 5:**
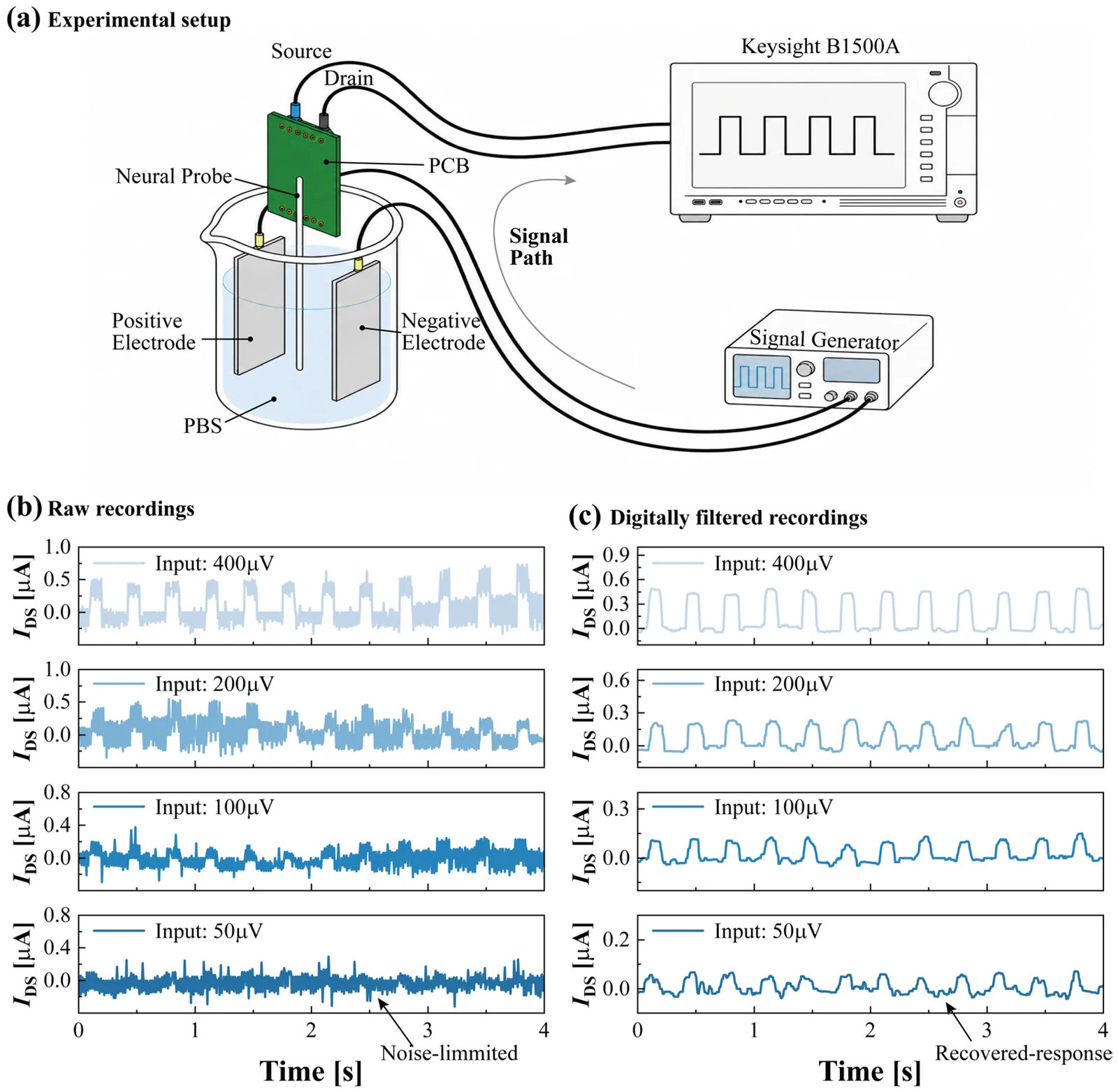
Reference-electrode-free analog signal recording: (**a**) Experimental setup in PBS; (**b**) raw source-drain current responses to 50–400 µV inputs; (**c**) corresponding responses after digital filtering.

Using the co-optimized device, we evaluated reference-electrode-free detection of weak analog neural signals. Analog inputs of 50, 100, 200, and 400 µV were applied to the sensing area using an external signal generator, while the source-drain current response was recorded with Agilent B1500A semiconductor parameter analyzer (Keysight Technologies, CA, USA). The measurement setup is shown in Figure 5a. Because small interfacial potential changes are converted into source-drain current modulation through transconductance, the source-drain current response was expected to scale with the input signal, as described by Equation (3).

(3) $$\Delta I_{DS}\approx g_{m}\cdot \Delta V_{signal}$$

With *V*_DS_ set to 0.6 V, the device produced stable current responses across the tested signal range, as shown in Figure 5b. At the lowest input amplitude of 50 µV, the stimulus-locked source-drain current response was not clearly distinguishable in the raw recording because it was masked by background instrumentation noise. After digital filtering, the time-locked response became clearly distinguishable, as shown in Figure 5c. The filtered 50 µV response showed a peak-to-peak amplitude of 67.83 nA, while the standard deviation of the filtered baseline noise was 26.89 nA, yielding a post-processed SNR of 8.04 dB. These results show that the optimized AlGaN/GaN HFET provides sufficient transconductance gain to detect microvolt-level signals without a reference electrode.

The noise observed in the raw recordings should be regarded as a system-level background rather than as intrinsic HFET noise alone. Potential contributors include noise from the external signal generator and transmission path, thermal noise from the HFET channel and measurement electronics, low-frequency 1/f noise associated with carrier and interface fluctuations in the transistor, and environmental electromagnetic interference coupled through the experimental setup. These noise components collectively increase the baseline fluctuation and therefore reduce the achievable SNR, with their influence becoming particularly evident for the 50 µV input. Because dedicated noise spectral-density measurements and component-wise noise characterization were not performed in the present study, the relative contribution of each noise source cannot be quantitatively separated. Accordingly, the reported 8.04 dB value should be interpreted as a post-processed system-level SNR under the present experimental conditions rather than as an intrinsic noise figure of the AlGaN/GaN HFET itself.

### 3.4. Ex Vivo Validation with Bullfrog Sciatic Nerve Recordings

**Figure 6:**
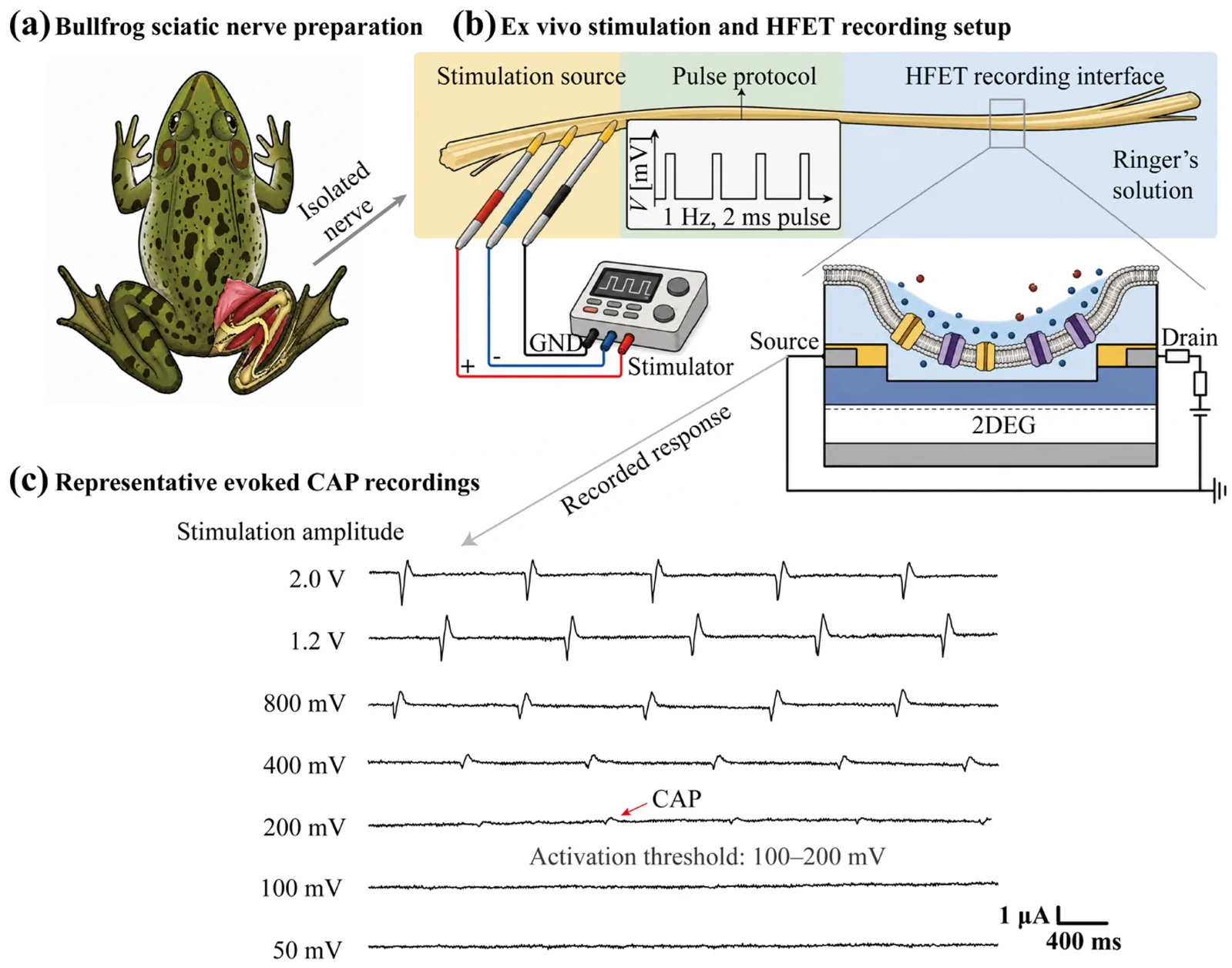
Ex vivo recording of bullfrog sciatic nerve activity: (**a**) Bullfrog sciatic nerve preparation. (**b**) Ex vivo stimulation and HFET recording setup. (**c**) Representative evoked CAP recordings under different stimulation amplitudes, indicating an activation threshold between 100 and 200 mV.

After confirming the probe response to controlled analog inputs, we further evaluated its ability to record biological electrophysiological signals using ex vivo bullfrog sciatic nerves. Before recording, nerve viability was verified by zinc–copper stimulation, and only preparations that exhibited clear muscle contractions were used. The sciatic nerve preparation and recording configuration are illustrated in Figure 6a and Figure 6b, respectively.

Pulsed electrical stimuli with a width of 2 ms and a frequency of 1 Hz were applied to the sciatic nerve. The stimulation amplitude was increased stepwise from 50 mV to 2 V, including 50, 100, 200, 400, and 800 mV, followed by 1.2 and 2 V. The stimulation signal was generated externally and delivered to the nerve through a stimulation probe, while the evoked neural response was recorded using Agilent B1500A semiconductor parameter analyzer (Keysight Technologies, Santa Rosa, CA, USA).

At stimulation amplitudes of 50 and 100 mV, neither muscle contraction nor characteristic neural response was detected, indicating that these stimuli were below the nerve activation threshold. When the stimulation amplitude was increased to 200 mV, clear compound action potentials (CAPs) were recorded after the stimulation artifacts, and stable muscle contractions were elicited. CAPs were also observed at higher stimulation amplitudes, as shown in Figure 6c. These results indicate that the activation threshold of the tested sciatic nerve lies between 100 and 200 mV. This threshold-dependent response is consistent with physiological neural recruitment and demonstrates that the probe can reliably record evoked neural activity. These ex vivo results further support the high sensitivity observed in the analog signal measurements.

To verify that the recorded responses originated from functional neural activity rather than nonspecific interference, repeatability tests were performed on the same nerve samples at 6 h intervals. Before the repeated measurements, zinc–copper stimulation no longer induced muscle contraction, indicating that the nerve had become inactive. Under the same electrical stimulation conditions, neither muscle contraction nor CAPs were detected. This absence of response confirms that the probe responds selectively to active neural tissue and does not produce false responses from inactive samples.

For active nerves, the CAP amplitude increased with stimulation intensity. The response progressed from no detectable signal below threshold to gradually larger amplitudes after activation, and finally approached a stable plateau at higher stimulation levels. This behavior is consistent with physiological recruitment in the bullfrog sciatic nerve and further supports the reliability and reproducibility of the recorded signals. Having established both microvolt-level signal detectability and ex vivo neural recording capability, the performance of the proposed HFET probe is further benchmarked against representative neural interface technologies below.

### 3.5. Comparison with Representative Neural Interface Technologies

To place the performance of the proposed AlGaN/GaN HFET probe in the context of existing neural interface technologies, Table 1 compares representative CMOS microelectrode arrays, polymer-based neural interfaces, electrolyte-gated transistors, nanoscale FETs, and AlGaN/GaN HFET sensors in terms of sensing architecture, signal-to-noise ratio, sensitivity or minimum detectable signal, and reported stability. Because the reported studies employ different device architectures, recording conditions, bandwidths, signal definitions, and post-processing methods, the numerical values should be interpreted as representative benchmarks rather than as a strict head-to-head comparison.

As summarized in Table 1, existing neural interfaces span a wide range of sensing architectures and performance characteristics. CMOS and polymer-based electrode systems provide mature electrophysiological recording capabilities, whereas transistor-based interfaces enable local semiconductor transduction directly at the sensing surface. The key distinction of the present device is therefore not simply the use of an AlGaN/GaN HFET for neural sensing. Instead, PEC recess engineering combined with sensing-region geometry optimization shifts the high-transconductance operating point toward zero gate bias, enabling reference-electrode-free self-biased operation while retaining microvolt-level signal detectability. The optimized device exhibits a peak transconductance of 32.47 mS/mm and resolves a 50 μV neural-like input with a post-filtered SNR of approximately 8.04 dB.

Short-term device stability was also examined over a period of several hours. During repeated electrical measurements over this period, no obvious degradation in the device characteristics or sensing response was observed. This observation indicates that the device maintained stable operation over the time scale investigated in the present study. Nevertheless, longer-term aging and chronic in vivo studies are still required to establish the stability of the proposed probe for chronic implantation.

## 4. Conclusions

This work demonstrates a reference-electrode-free AlGaN/GaN HFET neural probe enabled by photoelectrochemical recess engineering and sensing-region geometry optimization. The combined device-engineering strategy shifts the peak-transconductance operating point toward zero gate bias and increases the peak transconductance from 8.35 to 32.47 mS/mm, thereby enabling self-biased operation without an external reference electrode while maintaining high sensitivity to weak electrical signals. The optimized device resolves neural-like signals down to 50 µV, with a post-processed system-level SNR of approximately 8.04 dB under the present experimental conditions. Ex vivo bullfrog sciatic nerve experiments further demonstrate stimulus-dependent compound action potential recording, with responses observed above the neural activation threshold and disappearing after nerve inactivation. In comparison with representative neural interface technologies, the primary distinction of the proposed device lies in combining microvolt-level detectability with reference-electrode-free self-biased operation through direct engineering of the AlGaN/GaN sensing region. These results establish the feasibility of the proposed HFET architecture for neural electrophysiological interfaces.

## Figures and Tables

**Table 1:** Comparison of representative neural interface devices of biosensors.

Reference	Material (Architecture)	Reference Electrode	Metric	Stability	SNR
[12]	Pt (CMOS)	Required	µV	months	~4 dB
[24]	PEDOT:PSS (Parylene C)	Required	µV	days	>6
[25]	P3HT (EGOFET)	Required	mV	minutes	~60 dB
[3]	diF-TES-ADT (EGOFET)	Required	mV	-	-
[9]	p-type SiNW (BIT-FET)	Required	mV	hours	-
[27]	AlGaN (HFET)	Required	mV	-	-
This work	AlGaN (HFET)	Not required	µV	hours	~8.04 dB

## Data Availability

The raw data supporting the conclusions of this article will be made available by the authors on request.
